# More to ADHD than meets the eye: Observable abnormalities in search behaviour do not account for performance deficits on a discrimination task

**DOI:** 10.1186/1744-9081-1-10

**Published:** 2005-07-20

**Authors:** Edmund JS Sonuga-Barke, Sarah Elgie, Martin Hall

**Affiliations:** 1 Developmental Brain & Behaviour Unit, school of Psychology, University of Southampton, Highfield, Southampton, SO17 1BJ, UK

## Abstract

Children with Attention Deficit/Hyperactivity Disorder (ADHD) often perform poorly on tasks requiring sustained and systematic attention to stimuli for extended periods of time. The current paper tested the hypothesis that such deficits are the result of observable abnormalities in search behaviour (e.g., attention-onset, -duration and -sequencing), and therefore can be explained without reference to deficits in non-observable (i.e., cognitive) processes. Forty boys (20 ADHD and 20 controls) performed a computer-based complex discrimination task adapted from the Matching Familiar Figures Task with four different fixed search interval lengths (5-, 10-, 15- and 20-s). Children with ADHD identified fewer targets than controls (*p *< 0.001), initiated searches later, spent less time attending to stimuli, and searched in a less intensive and less systematic way (*p's *< 0.05). There were significant univariate associations between ADHD, task performance and search behaviour. However, there was no support for the hypothesis that abnormalities in search carried the effect of ADHD on performance. The pattern of results in fact suggested that abnormal attending during testing is a statistical marker, rather than a mediator, of ADHD performance deficits. The results confirm the importance of examining covert processes, as well as behavioural abnormalities when trying to understand the psychopathophyiology of ADHD.

## 

Attention Deficit/Hyperactivity Disorder (ADHD; [1]) is a disorder of childhood and adolescence characterised by a pattern of extreme, pervasive, persistent and debilitating inattention, overactivity and impulsiveness. Children with ADHD are more likely than their peers to experience educational under-achievement, social isolation and anti-social behaviour during the school years and to go on to have significant difficulties in the post-school years. Children with ADHD often perform poorly on tasks requiring the sustained and systematic allocation of attention over periods of extended time [2]. This appears to be true of tasks that require vigilance for rare targets amongst consecutively presented distractors (e.g., Continuous Performance Task; CPT – [3]). It is also true of more complex tasks that require self-directed and controlled search for targets amongst multiple concurrently presented distractors (e.g., Matching Familiar Figures Task; [4]).

In trying to explain the causes of this commonly observed pattern of performance deficit a range of different mechanisms operating at different levels of analysis have been invoked. For instance, cognitive accounts link deficits in performance to impairments in covert processes such as information encoding and retrieval as well as the 'holding in mind' of targets and their systematic comparison to distractors [5-7]. Such analyses fall firmly within the domain occupied by contemporary models of ADHD which emphasise its cognitive character [[8-10] for a discussion] and appear to receive considerable support from experimental studies of cognitive performance: ADHD children perform poorly on tasks thought to tap a range of executive and non-executive cognitive skills such as working and spatial memory, planning, attentional flexibility and inhibition [11-17].

Despite this strong body of evidence for the existence of cognitive deficits and the compelling nature of the cognitive deficit account, performance on complex discrimination tasks such as those described above can, in fact, be explained much more straightforwardly without invoking deficits in non-observable cognitive processes. This is because effective performance on these tasks requires the provision, protection and, systematic and skilled use of available processing time. This means one could account for the poor performance of ADHD children on such tasks purely in terms of their tendency to (i) start to attend later, and to terminate searches earlier – so producing a shorter duration of attention than controls (i.e., quantitative aspects of attending), and/or (ii) employ less systematic sequencing of attention to individual stimuli and to look at a smaller proportion of stimuli before trying to identify a target (i.e., qualitative aspects of search). Importantly if these quantitative and qualitative abnormalities in observed search behaviour exist they could affect the performance of ADHD children whether, or not, underlying, unobservable cognitive abilities are intact. Furthermore the theoretical significance of such a finding would be considerable. This is because it would in principle offer an explanation of poor performance on many laboratory tests; even those that have been used to index the non-observable cognitive processes discussed above. This in turn would caste doubt on the essentially cognitive nature of ADHD.

Given the potential significance of the search-based account of task performance it is surprising that there has not been more study of the quantitative and qualitative characteristics of search behaviour in ADHD. There is some evidence that children with ADHD fail to provide and exploit sufficient processing time during search. This appears to be partly because of difficulty either sustaining attention or modulating attentional fluctuations that occur over time or in protecting such time from interference by extraneous stimuli [3,18-20]. Furthermore Karatekin and Asarnow, [21] found that ADHD children initiated searches later than controls and fixated for shorter periods of time on more demanding tasks. Frank et al., [22] suggested that ADHD children's searches are self-terminating rather than exhaustive. However, there has been almost no enquiry into the structure of ADHD children's search or its level of organisation. Furthermore there has been no formal analysis of whether any abnormalities in the quantitative aspects of search described above in fact account for, or in statistical terms mediate, performance deficits in ADHD.

The study presented here compares the quality and timing of aspects of ADHD and control children's search behaviour while they are performing a computer-based fixed-trial version of the MFFT. The key questions were; (i) whether ADHD children differ from controls in these aspects of search behaviour and (ii) whether these differences in search behaviour, if identified, would mediate the association between ADHD and poor task performance. According to the statistical concept of mediation support for this hypothesis requires four predictions be confirmed.

I – that there is an association between ADHD and performance with children with ADHD performing more poorly than controls.

II – that there is an association between ADHD and search style with children with ADHD engaging in less systematic or efficient search behaviour than controls

III – that search-related style and behaviour are related to performance in the sample as a whole.

IV – that the association between ADHD and performance is lost when the association between ADHD and search style and behaviour is controlled.

In the current study we set out to test these predictions using 4 different fixed trial conditions in which children had 5-, 10-, 15- or 20 s to identify the target from amongst foils. These different time intervals were used to examine the extent to which ADHD and control children adapt their search style to different temporal constraints and to see whether the impact of restricted search behaviours becomes more important at particular search intervals.

In this study we used a novel approach to measuring search behaviour that did not employ eye gaze measurements. It was based on an analysis of different aspects of the timing and order in which initially 'covered' individual targets on the MFFT were accessed for viewing. The assumption in this study, therefore, is that targets accessed for inspection were actually inspected. This assumption could be tested in future studies using more traditional eye-tracking techniques.

## Results

### Do AD/HD children perform less well than controls?

The number of correct responses at each inspection interval is reported in Table 1 along with the *F *statistics and significance from a two-way ANOVA with group (ADHD v controls) as the between subjects factor and inspection interval (5-, 10-, 15-, 20-s) as the within subjects factor. There were effects of both inspection interval and group but no interaction between these factors. Controls outperformed children with ADHD at each level although these differences only reached significance at 5-, *t*(38) = -2.85; *p *< 0.01, 10-, *t*(38) = -3.22; *p *< 0.005 and 15-s, *t*(38) = -2.44; *p *< 0.05, but not at 20-s, *t*(39) = -1.18; *p *> 0.20. The significance of the quadratic term suggested that as one might expect children's performance demonstrated a diminishing level of gain for each additional 5 s of processing time. Introducing conduct or emotional problems as covariates into the analysis had no effect on this pattern of significance, F(1,36) = 13.84; p < 0.001.

**Table 1:** Performance and search characteristics of the ADHD and control groups.

		Mean Performance & Search Style (standard deviation)				F Statistics		
		5-s	10-s	15-s	20-s	Group	Interval	GxI

correct responses	Control	4.15 (2.21)	6.10 (1.68)	6.30 (2.20)	5.94 (1.84)	13.71*	13.61** 10.25**	0.6
	AD/HD	2.56 (1.13)	4.31 (1.80)	4.60 (2.26)	5.15 (2.36)			
number boxes open	Control	1.54 (0.37)	3.31 (0.50)	4.55 (1.04)	4.22 (1.14)	7.09*	138.42** 36.82**	5.12**
	AD/HD	1.30 (0.37)	2.51 (0.79)	3.42 (1.10)	4.14 (1.27)			
look time per trial (s)	Control	3.03 (0.24)	6.58 (0.68)	10.10 (1.50)	14.38 (1.47)	6.10*	548.9** *0.71*	1.54
	AD/HD	2.67 (0.47)	5.91 (1.11)	9.96 (1.45)	12.99 (2.71)			
search Initiation (s)	Control	1.49 (0.29)	1.58 (0.43)	1.61 (0.45)	1.65 (0.42)	4.85*	3.64* *1.77*	1.07
	AD/HD	1.59 (0.51)	2.02 (0.91)	1.99 (0.75)	2.00 (0.77)			
systematic searches	Control	5.00 (2.61)	7.95 (1.43)	7.46 (1.23)	7.62 (1.72)	25.76**	39.25** 36.07**	1.89
	AD/HD	2.35 (1.98)	5.20 (1.57)	6.15 (2.10)	5.52 (2.11)			

Note: AD/HD = Attention Deficit/Hyperactivity Disorder. Figures in parentheses are standard deviations. * p < 0.05; ** p < 0.01. Italicised figures in F column relate to quadratic contrasts.

### Was ADHD search behaviour less systematic, intensive and sustained than that of controls?

There was an effect of interval on all four search measures with the number of boxes opened, the length of time the boxes were looked at, the number of systematic searches and time at which searches were initiated all increasing as function of interval length. There was also an effect of group on all measures with ADHD children tending to begin searches later, open fewer boxes, look for less time overall and search in a less systematic way than controls. The effect of group persisted when conduct and emotional problems were entered as covariates; *F*(1,36) > 5.79; *p *< 0.05. The effect of group and interval interacted significantly only in the case of the number of boxes opened. This interaction was associated with a levelling off of the number searches made by children in the control group between the 15- and 20-s intervals. However, this interaction was much reduced when conduct and emotional problems were controlled, *F *(3,34) = 3.41; *p *< 0.05.

### Was AD/HD search style associated with poorer performance in the whole sample?

In order to address this question in an easily comprehensible way search style and performance variables were collapsed across search intervals (5-, 10-, 15- and 20-s). This was justified on the grounds that in general there appeared to be no interaction between inspection interval and group. This suggested that differences in search style and performance demonstrated between the groups were not systematically influenced by search interval (except for number of boxes opened). Furthermore interval specific measures of each variable showed good internal consistency. Cronbach's *α *for number of systematic searches (.81), number of boxes opened (.75), search initiation (.77), total look time (.55) and number of correct (.63) were all in the acceptable range. These aggregate scores for performance was significantly correlated with systematic searches (.30), number of boxes opened (.32), search initiation (-.42) and average look time (.34).

### Did search behaviour mediate the association between ADHD and poor performance?

For this analysis the number of search related-variables was reduced in order to simplify the test of the mediational hypothesis. The four measures were submitted to Principal Components Analysis with Oblimin rotation. The use of this technique for reducing the number of variables is justified on the basis of the current ratio of 1 item or search-related variable for every 10 subjects (i.e., 4 search variables and 40 subjects) although it needs to recognise that the solution may be unstable given the absolute size of the sample. Two factors with eigen values greater than 1 were extracted. These had the following loadings: Factor I (*intensive and systematic search*; 59.0 percent of the variance) had loadings of .83 for number of systematic searches, .98 number of boxes opened, -.43 for search initiation and -.14 for average look time. The loadings for Factor II (*late start – short look*; 27.6 percent of the variance) were -.17 for number of systematic searches, .15 number of boxes opened, .70 for search initiation and -.99 for average look time.

**Figure 1:**
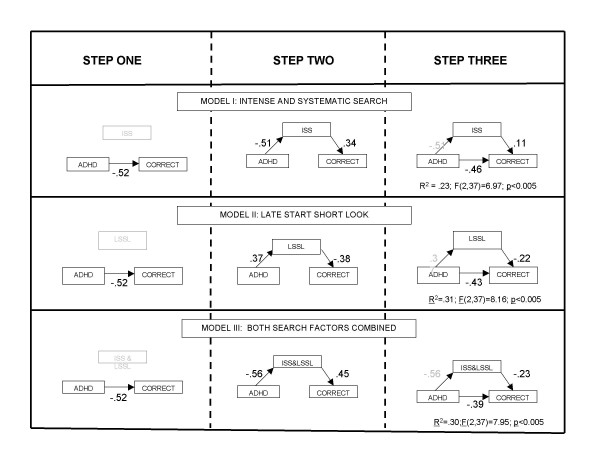
Diagrammatic representation of the three steps of the mediational analysis of the effects of search style on performance. In each case step one involves the regression on ADHD group status on to the number of correct responses: Step two involved regressing ADHD into the search factor and then the search factor score on the number of correct responses: Step three involved simultaneously regressing ADHD status and search factor scores onto the number of correct responses. ISS = intense and systematic search; LSSL = Late start short look.

The mediational hypothesis was tested using a standard three stage procedure using single and multiple regressions for each search factor separately and with both search factors together. In stage one ADHD status was regressed onto the number of correct responses and the standardised *β *coefficient derived indicating the strength of the association. In stage 2 the completeness of the chain of associations linking ADHD to performance via search factors was tested by regressing ADHD status onto search behaviour and search behaviour onto the number of correct responses. In stage 3 the search factor was introduced alongside ADHD into a multiple regression analysis to test whether the pathway between ADHD and performance remained significant once search behaviour was controlled. ADHD status was entered in step 1 on the model and search behaviours in step 2. The results are presented diagrammatically in figure 1 along with the regression statistics. As indicated by the preceding analyses ADHD is related to performance and search behaviour. Also search behaviour is related to performance for both search factors and their combination. However, the association between ADHD and performance, although slightly but not significantly reduced, remained clearly significant when search behaviour and ADHD were introduced alongside each other into the analysis. In fact in each case it was the link between search behaviour and performance that was reduced to non-significant levels. This suggests that this association was partly the result of a common effect of ADHD status on both search behaviour and performance. These effects were not affected by entering conduct and emotional problems into the analysis alongside ADHD status into the multiple regression analyses.

## Discussion

The results of the current are consistent with the previous evidence that ADHD children perform poorly on complex tasks requiring systematic search. They also provide new evidence about the abnormalities in their style of search. However, they give no support to the assertion that their performance on such tasks is the result of abnormalities in observable characteristics of their search behaviour; either in terms of the reduced amount of time allocated to search, because of a late onset and a premature off-set of searching, or the more chaotic and sluggish search style. Even taking into account these factors ADHD children still performed more poorly than controls. While it is possible that aspects of search-related behaviour other than those observed and coded might have been the constraining element it is difficult to identify what these additional characteristics might have been. It is also possible the approach taken to measuring search behaviour was insensitive to more subtle differences between the groups that might become obvious if more fine grained approaches to measuring search such as eye-tracking were used. However, one possibility is that deficits in performance displayed by the children with ADHD were the result of deficits in non-observable processes that either were unrelated to search behaviour or at least did not have their effect on performance via an impact on search behaviour. In this sense the current data add support to the idea that ADHD is a disorder with distinct cognitive and behavioural elements which will probably affect different domains of functioning in different ways. From a practical point of view this suggests that interventions targeted at improving attending or search behaviour rather than improving underlying processes are unlikely to be successful in improving task performance.

The performance data is interesting in itself for a number of reasons. First, it does not provide support for accounts of ADHD performance on tasks of extended duration that stress the role of premature task disengagement and the existence of a deficit in sustained attention [23]. Such accounts would have predicted an interaction between group and inspection interval with errors increasing across interval duration at a greater rate in the ADHD, than the control group. In contrast to this prediction the size of the deficit displayed by children with ADHD, relative to controls, was largely independent of interval length. In fact deficits were most marked on the 5-, 10- and 15-s intervals, the opposite of what would be predicted by a task disengagement account. At first glance, this seems to suggest that, relative to that of controls, ADHD performance improved on the 20-s trial. A closer inspection of the results reveals that the narrowing gap between the two groups on this interval is due as much to a slight decline in the control performance as it is to improvements in the ADHD group. Second, the results provide no support for the state regulation account of ADHD [24]. According to this account ADHD children's difficulties arise because of problems modulating their energetic state to meet the changing demands of different settings [25]. A key prediction from this account is that the performance of children with ADHD will be disrupted in settings and tasks with either high (over-arousing) or low (under-activating) rates of stimulus presentation. Support for the state regulation account recently came from a paper reporting the results of two experiments using a similar version of the MFFT to that employed in the current study [26]. In these studies a quadratic interaction between ADHD group status and inspection interval was reported (5-, 10-, and 15-s) with ADHD children performing less well than controls on 5- (short) and 15-s (long) intervals but as well as them on 10-s intervals. The failure of the current study to replicate this pattern may be due to differences between the studies in terms of task difficulty. For instance, in the task version used in the Sonuga-Barke [26] paper all test stimuli were visible throughout every trial as in the original MFFT. In the version used in the current only one stimulus was visible at any one time. The current task was therefore more likely to tap working memory capacities more directly as participants had to hold one stimulus in mind if they wanted to compare it directly with another. The task used in the current paper was therefore likely to have a higher cognitive load and be considerably more demanding than the task used previously. This suggestion is confirmed by a direct comparison of performance on the two tasks. The average error rate in the current study was 54 percent while in the previous studies it was 43 and 44 percent respectively (*F*(1,78) = 8.85; *p *< 0.001). It is possible that in the current study the increased levels of task difficulty masked cognitive-energetic effects associated with interval duration. A direct comparison of the two versions of the MFFT is required to test this hypothesis. There was no evidence to support the view that children with AD/HD produce better than expected performance on shorter trials (i.e., 5-s intervals) because they employ compensatory strategies developed in response to the impact of their own impulsive cognitive style on processing opportunities [26]. Deficits were as great on the 5-s, as they were on the 10-, 15- and 20-s, intervals.

The data on search behaviour are also interesting in its own right. ADHD children took longer to initiate their search and spent less time attending to stimuli during the task. Second, they tended to be less systematic and slower during searches when they were actively on task. These findings are consistent with the impression given by previous studies of ADHD children search-related performance [21,22,27]. However, they do not fit well with accounts of ADHD that emphasize the impulsive and disinhibited nature of ADHD children's cognitive performance [28]. On the current task ADHD children's performance was marked by a slow start to searching, a longer time to look at each stimuli and a slower passage from one stimulus to another. This is consistent with the view that ADHD children process information more slowly, rather than more quickly, than controls [29]. However, it must be born in mind that the task used in this study was externally paced, rather than, self-paced. Trial length could not be shortened by 'impulsive responding', a factor that appears to be crucial in determining the extent to which children with ADHD are willing to trade accuracy for speed [30].

The difference between the groups in terms of the ISS factor was considerable for the 5-, 10-, and 15-s intervals. However, there was no difference between the groups at the 20-s interval value. This was related to a drop in the levels of ISS displayed by controls rather than an increase by children with ADHD. This decrease in ISS by the controls was largely due to a slowing down of the average rate of search rather than a drop in other elements of the ISS construct (e.g., numbers of systematic searches). Such a change is perhaps not surprising given the fact that rapid search becomes less important on longer trials. In fact slowing search down could be regarded as a sign that controls were able to adapt their search behaviour to the existing time constraints.

## Method

### Participants

Forty boys between the ages of eight and twelve (20 ADHD and 20 typically developing controls) took part in the study. The ADHD children were recruited through National Health Service child psychiatric and paediatric outpatient clinics. All had a diagnosis of Hyperkinetic Disorder [31] the equivalent of severe combined type ADHD which affects approximately only 30 percent of the most severe ADHD cases [32]. Diagnosis was made against research criteria on the basis of a thorough clinical investigation that involved parental and child interviews, parent and teacher rating scales and direct observation of the child. In addition, children were only included in the ADHD group if they scored above the clinical cut-off (seven or more out of ten) on the hyperactivity scale of both the parent and teacher versions of the Strengths and Difficulties Questionnaire [SDQ; [33]].

The SDQ is a development of the Rutter questionnaire [34]. It has an extended hyperactivity scale and scales measuring more general conduct and emotional problems [35]. The psychometric properties and norms of the SDQ have been extensively examined in large epidemiological studies within the pre-school age group [36]. Together these criteria would place the ADHD children included in the current sample within the top two or three percent of the childhood population in terms of severity of ADHD symptoms and impairment. No child in the ADHD group received a diagnosis of Hyperkinetic Conduct Disorder although many showed some signs of conduct problems (see table 2). Children were identified from a region of predominantly Caucasian ethnic composition that had a mixed socio-economic background. Seventy-five boys with a diagnosis were originally identified, 35 of these agreed to take part in the study. Fifteen children from the original group were excluded because they did not meet entry criteria for the study (i.e., they had an IQ below 80 and/or were outside the required age range). Children refrained from taking any medication prescribed for ADHD in the 24 hours prior to testing. Controls were selected at random from local schools that reflected the ethnic and socio-economic composition of the region in general. They were children who did not meet either parent or teacher borderline cut-offs for hyperactivity on the SDQ (i.e., less than six on both parent and teacher scales; [33]). Of the 50 control children who were initially contacted 26 agreed to participate. Four of these children were excluded because they failed to meet the inclusion criteria for controls. Two children were excluded because they failed to co-operate during testing.

**Table 2:** Age, IQ and behaviour ratings for the ADHD and control children.

	CONTROLS	AD/HD	
Mean age	9.9 (0.7)	10.5 (1.5)	t = 1.65
Mean IQ	110.3(17.8)	106.7 (13.1)	t = 0.71
% conduct problems	0	35	χ^2 ^= 8.48**
% emotional problems	0	20	χ^2 ^= 4.44*

Note: AD/HD = Attention Deficit/ Hyperactivity Disorder; * p < 0.05; ** p < 0.01; % behaviour problems based on children meeting SDQ criteria for presence of difficulties at home and school. Figures in parentheses are standard deviations.

IQ was measured using four sub-scales of the Wechsler Intelligence Scale for Children (WISC-IIIR; [37]); similarities, vocabulary, block design, object assembly. The four sub-tests were pro-rated and an estimate of the child's full scale IQ was derived. Table 2 shows the mean IQ scores and ages for members of the two groups as well as the proportion of children who met SDQ cut-offs for borderline conduct or emotional problems. While groups did not differ in terms of either age or IQ the ADHD group had a higher proportion of children with conduct and emotional problems. Analyses were performed both with and without behavioural and emotional problems as covariates.

### Procedure

Children took part in one session consisting of four blocks of 10 trials. At the start of each trial the target stimulus was presented in a box measuring 5.5 × 6.5 cm in the centre at the top of the computer monitor screen. Six other boxes of the same dimensions were presented in two rows of three under the target stimuli. These contained the six test stimuli (five foils and a second copy of the target stimulus). The position of the target copy was varied randomly amongst the foils. At the start of each trial all six boxes containing test stimuli were 'covered' by a white square equal in size to the surrounding box. The target stimulus was visible throughout the inspection period of each trial. To 'open' a box to view one of the six test stimuli participants were required to click on the area within the box with a mouse controlled cursor. When participants had finished inspecting the stimuli in the open box a second click closed the box and re-covered the stimuli. Participants could not open a second or subsequent box until they had closed the box they were inspecting. In block one, children had 5-s in which to inspect the stimuli on each trial, in block two the viewing time was 10-s, in block three it was 15-s and in block four it was 20-s. The order of presentation of these blocks was completely randomised across subjects. At the end of the inspection period (5-, 10-, 15- or 20-s) the squares 'covering' the stimuli were removed so that all stimuli were visible. At the same time the target was covered in order to remove the opportunity for children to continue to search after the end of the viewing period. The participants were prompted to identify the copy of the target by the words "choose now" written on the computer screen. This was achieved by moving the cursor over the chosen stimulus and pressing the button on the top of the mouse. Children were allowed one attempt to select the target on each trial. The experiment was presented on, and data collected using, a Pentium 75 computer connected to a mouse. Variables collected included; time to initiation of search; number of boxes 'opened' during each trial; order in which boxes were opened and length of time which boxes were opened. Two higher order variables were derived: The number of exhaustive searches (i.e., the number of times all boxes were opened during a trial) and the number of systematic searches. A search was deemed to be systematic when boxes were opened in a systematic order (i.e., going across columns or up or down rows) with no box being opened twice until all other boxes had been opened. Ratings of these variables were extremely reliable with 100 percent agreement between two raters. All sessions were video taped and periods of on- and off-task behaviour was recorded. Computer generated, synchronised, real-time information about search behaviour (i.e., if boxes were open or not at a particular point in time) was printed on to the video tape of each session so that the time open boxes were being attended to could be estimated. Inter-rater reliability for these estimates was high with 98 percent agreement between raters.
